# Outbreak of *Serratia marcescens* in a neonatal intensive care unit at a Pretoria hospital

**DOI:** 10.4102/safp.v68i1.6353

**Published:** 2026-10-06

**Authors:** Adeline M. Nkoane, Sunday O. Okeke, Doudou K. Nzaumvila, Tombo Bongongo

**Affiliations:** 1 Department of Family Medicine and Primary Health Care, School of Medicine, Sefako Makgatho Health Sciences University, Pretoria, South Africa

**Keywords:** *Serratia marcescens* outbreak, NICU, Jubilee District Hospital, Pretoria, South Africa

## Abstract

**Background:**

An outbreak of *Serratia marcescens* disrupted care in a neonatal intensive care unit (NICU) at Jubilee District Hospital in Pretoria, South Africa. This study profiled infected neonates and identified outbreak factors.

**Methods:**

Retrospective review of an epidemiological outbreak of *S. marcescens* in the NICU of Jubilee District Hospital between 2022 and 2023.

**Results:**

From January 2022 to December 2023, 273 neonates with *S. marcescens* were admitted. Most were male (56.27%), under 1 day old at admission (68.86%), and had low birth weight (59.27%). Admissions mainly came from the hospital’s labour ward (70.61%). *Serratia marcescens* (95.97%) was the predominant bacteria, with *Klebsiella pneumoniae* (4.03%) less common. Mortality was 16.12%. Multivariable analysis showed low birth weight (OR = 6.16, 95% CI: 2.27–16.70; *p* < 0.001) and age at first blood culture (OR = 2.98, 95% CI: 1.78–4.98; *p* < 0.001) independently increased mortality risk.

**Conclusion:**

A *S. marcescens* outbreak in the NICU of Jubilee District Hospital between January 2022 and December 2023 revealed that *S. marcescens* mainly affected low-birth-weight male neonates admitted within 1 day of the labour ward. Low birth weight and early blood culture timing were linked to higher mortality. These results highlight the urgent need to enhance infection control in the labour ward, update and enforce policies that protect vulnerable neonates, and ensure timely blood culture testing and treatment to reduce mortality.

**Contribution:**

The Jubilee NICU *S. marcescens* outbreak affected mainly low-birth-weight male neonates admitted within 1 day from the labour ward. Low birth weight and early blood culture timing increased mortality risk. These findings highlight the need to strengthen infection control in labour wards, protect vulnerable neonates, and ensure timely blood culture and treatment to reduce deaths.

## Introduction

*Serratia marcescens*, a Gram-negative bacterium first identified in 1819 by Italian pharmacist Bizio,^1^ poses a significant threat to preterm newborns, particularly those in neonatal intensive care units (NICUs). These infants are especially vulnerable to bloodstream infections due to common medical interventions such as intubation, catheterisation, or total parenteral nutrition.

In the United States, bloodstream infections are notably prevalent among newborns with low birth weight (under 1500 g), those requiring mechanical ventilation, and those with central catheters.^2^ Key risk factors include prematurity, medical interventions such as mechanical ventilation, and the insertion of central venous or umbilical catheters, which have been associated with an increased risk of late-onset sepsis (LOS) caused by *S. marcescens*. Prolonged hospitalisation and stays in the NICU further increase exposure to nosocomial pathogens. Additionally, the use of broad-spectrum antibiotics, including empirical treatments, can disrupt normal flora, allowing opportunistic pathogens to thrive.^3^ Further studies have highlighted that the underdeveloped immune system of preterm infants significantly heightens their vulnerability to infection.^4^

Neonatal sepsis is a bloodstream infection occurring within the first 28 days of life and is classified as early-onset sepsis (EOS) or LOS. Early-onset sepsis arises within the first 48–72 h of life and is typically caused by pathogens transmitted vertically from the mother before or during delivery. In contrast, LOS develops after 72 h in term infants and can result from both vertical and horizontal transmission.^5^ Numerous studies emphasise that cross-infection in NICUs is a major contributor to neonatal sepsis. While EOS is mainly linked to maternal transmission, LOS is more often associated with hospital-acquired infections or invasive medical procedures.^6^

Early diagnosis and prompt antibiotic therapy are crucial to reducing morbidity and mortality associated with EOS. Empirical antibiotic treatment should be administered without delay to neonates suspected of infection to ensure effective management of neonatal sepsis.^7^

In Spain, a study confirmed that the primary issue that preterm infants encounter while in NICUs is nosocomial sepsis. Although *S. marcescens* is a new cause of preterm sepsis, not much is known about its prevalence. The team highlighted the rapid colonisation of the gut by *S. marcescens* in preterm infants, especially those born before 30 weeks of gestation and weighing less than 1500 g.^8^ This early colonisation is concerning due to several factors, such as gut microbiota and disease susceptibility. Recent research suggests that alterations in the gut microbiota of preterm infants can precede the development of severe conditions such as sepsis and necrotising enterocolitis.^8^ Early colonisation by pathogens like *S. marcescens* can disrupt the normal microbial balance, increasing vulnerability to this infection.^9^

In Cape Town, South Africa, Enterobacterales resistant to carbapenems pose a significant health risk due to high mortality rates. Understanding the clinical and microbiological epidemiology of this resistance is crucial for improving infection prevention, antimicrobial management, control, and therapy for both new and current antibiotics.^2^ The study revealed that 90% of participants, aged 7 days to 88 years, were from public hospitals. Factors such as recent antibiotic exposure, medical device use, and surgery were found to be significant risk factors for *S. marcescens* infection. The most common species in Cape Town was *Klebsiella pneumoniae*.^2^ While educating Pretoria’s healthcare professionals, this study intends to profile the affected neonates admitted to Jubilee District Hospital’s NICU between January 2022 and December 2023, and to uncover the factors linked to the outbreak.

## Research methods and design

### Study design

This was a retrospective record review of patients with *S. marcescens* in the NICU at Jubilee District Hospital from 2022 to 2023.

### Setting

The study was carried out in the NICU of Jubilee District Hospital located in the Hammanskraal area in the northern area of Gauteng province, South Africa. The site is located along the N1 national road, approximately 50 km from Pretoria’s Centre. This is the only district hospital in Region 2 of the Tshwane Health District. Jubilee District Hospital is classified as a rural hospital and began as a missionary hospital almost 60 years ago. The main hospital has 551 beds, and another 300 beds were added as part of the Alternative Building Technology (ABT) structure established during the coronavirus disease 2019 (COVID-19) pandemic. The hospital serves patients from Hammanskraal and the surrounding districts, which include provinces such as Northwest, Mpumalanga, and Limpopo. It serves 32 clinics (21 clinics in the North West Province and 11 clinics in Gauteng). The hospital employs 53 general practitioners or medical officers and seven medical specialists (two family physicians, one anaesthesiologist, one paediatrician, two obstetricians, and one orthopaedic surgeon).

### Study population, sampling strategy, and sample size

All medical records of neonates diagnosed with *S. marcescens* in the NICU at Jubilee District Hospital from January 2022 to December 2023 were considered in this study. The hospital’s NICU logbook revealed that 273 patients tested positive for *S. marcescens* while in the NICU during the specified period. The medical record was also chosen based on the inclusion criteria, including diagnosis, age at admission, gender, birth weight, date of blood culture collection, result of blood culture, patient provenance (from Jubilee Maternity or outside of Jubilee Hospital), and treatment outcome. Convenience sampling was used, and the sample size was 273 neonates (*N* = 273).

### Data collection

After receiving ethical clearance, data collection was completed in the first half of January 2025. All the neonate files from January 2022 to December 2023 were obtained by a research assistant who was trained by the principal investigator. Only files with the diagnosis of *S. marcescens* were included in the population study. The study sample was limited to those for whom research data were available, including diagnosis, file number, age, patient proof (home, labour ward, etc.), treatment, and case outcome. A second research assistant, also trained by the principal investigator but not in contact with the first one, cross-checked the different data collection steps to ensure that the procedure yielded comparable results.

### Data analysis

The raw data were captured, cleaned, and coded from Microsoft Excel and subsequently imported into Stata-18 (Stata Corp., College Station, Texas, US) for analysis. Missing data were handled using an available-case approach, where analyses for variables with incomplete information included only records with available data. As a result, the denominator varied across variables. For example, age at admission was recorded for 263 patients and sex for 262 patients, out of a total of 273. Percentages were calculated based on the number of observations available for each variable. The socio-demographic data were analysed descriptively and presented using frequency tables. Bivariate analysis was conducted using Fisher’s exact test to identify which factors were significantly associated with fatality, using a confidence interval of 95% and a *p*-value of 0.05. Where necessary, numeric variables were converted to categorical data to meet statistical testing standards. Variables significantly associated in bivariate analysis were included in the final binary logistic regression model to identify which factors remained significant (*p* ≤ 0.05).

### Ethical considerations

Permission to conduct the study was obtained from the Chief Executive Officer of Jubilee District Hospital, with clearance given by the Sefako Makgatho University Research Ethics Committee (SMUREC/M/343/2024: IR). Anonymity was observed during the entire research process.

## Results

### Socio-demographics

The sample consisted of 273 neonate cases. Most of them were males (*n* = 148; 56.27%) who were less than a day old (*n* = 188; 68.86%) and had a low birth weight (*n* = 162; 59.27%). Among the episodes of bacteraemia, a large proportion of infected neonates constituted hospital-acquired cases (180; 70.6%), whereas a few (39; 14.8%) were home-acquired cases. There was a case fatality rate of 19%, as approximately 44 (16.12%) neonates died. Further details are provided in Table 1.

**TABLE 1:** Socio-demographic characteristics of the neonates with *Serratia marcescens*.

Variable	*n*	%	Mean	s.d.	Min	Max
**Age (days) at admission (*n* = 263)**	-	-	2.34	6.05	0	39
< 1	188	68.86	-	-	-	-
1–3	26	9.52	-	-	-	-
4–6	23	8.42	-	-	-	-
≥ 7	26	9.52	-	-	-	-
**Sex (*n* = 262)**						
Male	148	56.27				
Female	114	43.35				
**Birth weight (*n* = 273)**	-	-	2226.56	810.71	640	4590
Low birth weight	162	59.34	-	-	-	-
Normal birth weight	108	39.56	-	-	-	-
Overweight	3	1.10	-	-	-	-
**Admitted from (*n* = 263)**						
Jubilee	185	70.61	-	-	-	-
Home	39	14.89	-	-	-	-
Other healthcare facilities	38	14.50	-	-	-	-
**Age (days) at the time of blood culture (*n* = 273)**	-	-	7.18	6.85	0	44
< 1	4	1.47	-	-	-	-
1–3	65	23.81	-	-	-	-
4–6	102	37.36	-	-	-	-
≥ 7	102	37.36	-	-	-	-
**Organism (*n* = 273)**						
*K. pneumoniae*	11	4.03	-	-	-	-
*S. marcescens*	262	95.97	-	-	-	-
**Outcome of the patient (*n* = 273)**						
Demised	44	16.12	-	-	-	-
Discharged	178	65.20	-	-	-	-
In the ward	43	15.75	-	-	-	-
Other	8	2.93	-	-	-	-
**Case fatality rate**		19.38				
Fatal	44	16.12	-	-	-	-
Non-fatal	229	83.88	-	-	-	-

s.d., standard deviation.

### Factors associated with fatality

Fisher’s exact tests were used to determine factors that were significantly associated with fatality (*p* ≤ 0.05). It was found that three factors were associated with fatality: Age at admission, birth weight, and age at first blood culture. Higher proportions of case fatality were significantly associated with neonates aged 3 days or younger at admission and at the time of blood culture, as well as those with low birth weight. Further details are provided in Table 2.

**TABLE 2:** Factors associated with fatality.

Factor	*n*	%	Case fatality				Fisher’s exact test
			Fatal		Non-fatal		
			*n*	%	*n*	%	
**Age (days) at admission**	-	-	-	-	-	-	0.005*
≤ 3	224	82.05	43	97.73	181	79.04	-
4–6	23	8.42	1	2.27	22	9.61	-
≥ 7	26	9.52	0	0.00	26	11.35	-
**Sex**	-	-	-	-	-	-	0.313
Male	148	56.27	20	46.51	128	58.18	-
Female	114	43.35	23	53.49	91	41.36	-
**Birth weight**	-	-	-	-	-	-	0.000*
Low birth weight	162	59.34	39	88.64	123	53.71	-
Normal birth weight	108	39.56	5	11.36	103	44.98	-
Overweight	3	1.10	0	0.00	3	1.31	-
**Age (days) at the time of blood culture**	-	-	-	-	-	-	0.000*
≤ 3	69	25.27	24	54.55	45	19.65	-
4–6	102	37.36	16	36.36	86	37.55	-
≥ 7	102	37.36	4	9.09	98	42.79	-
**Organism**	-	-	-	-	-	-	0.693
*K. pneumoniae*	11	4.03	2	4.55	9	3.93	-
*S. marcescens*	262	95.97	42	95.45	220	96.07	-

* , Fishers exact test *p*-value < 0.05; Non-fatal, discharged or in-ward or other.

### Multivariate logistic regression

The significant factors from Fisher’s exact test were utilised to build a binary logistic regression model, and only two variables remained significant (*p* ≤ 0.05). Neonates between the ages of 0–3 days had 3.13 times greater odds of fatality (*p* ≤ 0.05); similarly, neonates with a low birth weight had a 6.12-fold increased likelihood of fatality. Table 3 shows details of the logistic regression model.

**TABLE 3:** Factors linked to the outbreak.

Factors	Odds ratio	Std error	*P* > |*z*|	95% CI	
				Lower limit	Upper limit
Age (days) at admission	3.129044	2.754889	0.195	0.5571761	17.57239
Birth weight	6.155981	3.134416	0.000*	2.269313	16.69937
Age (days) at the time of blood culture	2.980697	0.7809166	0.000*	1.783653	4.981099

* , Binary logistic regression: *p* < 0.05; Std error, standard error; CI, confidence interval.

## Discussion

*Serratia marcescens* has emerged as a significant cause of nosocomial infections, particularly in NICUs.^1,7^ This was the case in the newborn intensive care unit of Jubilee Hospital in Pretoria, South Africa, with most infected neonates (70.6%) originating from the same facility, as shown in Table 1. This is in line with what has been identified in the literature as the main source of infection. Due to this, researchers were asked to investigate microbial behaviour in hospital ward environmental reservoirs by the Division of Healthcare Promotion at the Centres for Disease Control and Prevention.^2^ The hospital environment, which includes surfaces and drains, is becoming more widely acknowledged as a reservoir for bacterial species, clones, and plasmids of significant epidemiological importance in connection with *S. marcescens* epidemics.^3^ Outbreaks are most likely the result of favourable environmental circumstances and gaps in infection protection procedures within healthcare facilities.^10^

In the current study, the predominant bacteria present in the Jubilee District Hospital NICU were *S. marcescens* (96%), followed by *K. pneumoniae* (3.9%), as presented in Table 2. However, the literature lists several bacterial species, such as *Acinetobacter baumannii, Escherichia coli, K. pneumoniae, Pseudomonas aeruginosa, Staphylococcus aureus*, and *S. marcescens*, that can result in an epidemic in a NICU. In a study carried out in Madrid, Spain, it was discovered that the prevalences of *S. marcescens* and *K. pneumoniae* were similar to those in Pretoria, South Africa, with *S. marcescens* at 96% and *K. pneumoniae* at 4%.^8^ Genetic testing for multidrug resistance to *S. marcescens* was carried out in some of the studies pertaining to the outbreaks, including in South Africa.^2,9^ The current study did not follow that course, possibly because of the limited funding available for the investigation. Nosocomial *S. marcescens* outbreaks in neonatology units are difficult to control; they require a multidisciplinary approach with strict infection prevention measures to mitigate risk factors.^10^

In the Jubilee District Hospital study, low birth weight (< 1500 g) emerged as the primary factor associated with the acquisition of *S. marcescens* infection. This finding aligns with those of the study conducted by Cristina et al., which also identified low birth weight and immune system immaturity in preterm newborns as key risk factors for developing Serratia infection.^11^

In the Jubilee study, more than half of the patients were discharged (65%); those who died from the Serratia infection made up about 16%. A systematic study conducted by Zivkovic et al. found nearly identical treatment outcomes, with roughly half of the patients recovering entirely (50%), compared with a death rate of less than 50%.^12^

Multivariable logistic regression analysis identified low birth weight and age at first blood culture as independent predictors of mortality among neonates with bloodstream infections. Neonates with low birth weight had approximately six-fold higher odds of mortality compared with those with normal birth weight. This finding is consistent with previous studies that have identified low birth weight as an important determinant of adverse neonatal outcomes and mortality.^13,14^ Low birth weight infants are particularly vulnerable to severe infections because of their immature immune systems, reduced physiological reserves, and increased susceptibility to complications associated with prematurity.^15^ Recent evidence has similarly demonstrated that low birth weight and prematurity are major contributors to poor outcomes among neonates with sepsis and bloodstream infections.^16^ Furthermore, neonates whose first blood culture was obtained within the first 3 days of life had nearly three-fold higher odds of mortality. This finding suggests that severe infections occurring early in life are associated with poorer outcomes, likely reflecting EOS, which has been consistently linked to increased morbidity and mortality.^16^

This study employed a descriptive cross-sectional design through retrospective record review, allowing for the systematic collection and analysis of medical records based on predefined criteria. However, the absence of genetic investigations, which could have been integrated into ongoing genomic surveillance efforts, limited the study’s scope. Such surveillance is essential for enhancing infection prevention, control, and antimicrobial stewardship. The findings highlight an urgent need to strengthen infection control measures within healthcare facilities – particularly in labour wards – and to implement policies and practices that protect vulnerable neonates.

## Conclusion

The outbreak in the Jubilee NICU, between January 2022 and December 2023, revealed that *S. marcescens* primarily affected low-birth-weight male neonates admitted within 1 day from the labour ward. Both low birth weight and early timing of blood culture were associated with higher mortality. These findings highlight the urgent need to strengthen infection control in the labour ward, implement targeted bacterial surveillance, prioritise care for vulnerable neonates, and ensure timely blood culture and treatment to reduce mortality.
